# Seroepidemiology of Parechovirus A3 Neutralizing Antibodies, Australia, the Netherlands, and United States 

**DOI:** 10.3201/eid2501.180352

**Published:** 2019-01

**Authors:** Eveliina Karelehto, Lieke Brouwer, Kimberley Benschop, Jen Kok, Kerri Basile, Brendan McMullan, William Rawlinson, Julian Druce, Suellen Nicholson, Rangaraj Selvarangan, Christopher Harrison, Kamani Lankachandra, Hetty van Eijk, Gerrit Koen, Menno de Jong, Dasja Pajkrt, Katja C. Wolthers

**Affiliations:** Academic Medical Center, Amsterdam, the Netherlands (E. Karelehto, L. Brouwer, H. van Eijk, G. Koen, M. de Jong, D. Pajkrt, K.C. Wolthers);; National Institute for Public Health and the Environment, Bilthoven, the Netherlands (K. Benschop);; Institute of Clinical Pathology and Medical Research, Westmead, New South Wales, Australia (J. Kok, K. Basile);; Sydney Children's Hospital, Sydney, New South Wales, Australia (B. McMullan);; Prince of Wales Hospital, Randwick, New South Wales, Australia (W. Rawlinson);; Doherty Institute, Melbourne, Victoria, Australia (J. Druce, S. Nicholson);; Children’s Mercy Hospital, Kansas City, Missouri, USA (R. Selvarangan, C. Harrison);; Truman Medical Center, Kansas City (K. Lankachandra)

**Keywords:** Picornaviruses, parechovirus A3, outbreak, neutralizing antibody, seroprevalence, seroepidemiology, viruses, Australia, the Netherlands, United States

## Abstract

Recent parechovirus A3 (PeV-A3) outbreaks in Australia suggest lower population immunity compared with regions that have endemic PeV-A3 circulation. A serosurvey among populations in the Netherlands, the United States, and Australia before and after the 2013 Australia outbreak showed high PeV-A3 neutralizing antibody prevalence across all regions and time periods, indicating widespread circulation.

Parechovirus A3 (PeV-A3), belonging to the Picornavirus family, can cause respiratory and gastrointestinal symptoms, as well as meningitis and sepsis-like disease in infants (*1*). PeV-A3 was isolated from a fecal specimen collected in 1999 from a child with fever, diarrhea, and transient paralysis; it has been gaining increasing interest because of reported outbreaks of severe illness in neonates (*2**–**4*). To date the largest outbreaks have been caused by a recombinant PeV-A3 strain in Australia: in New South Wales in 2013, and in Victoria in 2015 (*4*). Humoral immunity is essential in protection against PeV-A3 disease, yet seroepidemiological data on population immunity are limited (*5*,*6*). We describe the findings of a cross-sectional study on serum PeV-A3 neutralizing antibody (nAb) levels among children and adults from Victoria and New South Wales, Australia; Missouri, USA; and the Netherlands, where PeVs circulate every 2 years during summer and fall months (*3**,**7*).

## The Study

We screened 1,288 anonymized serum samples from persons 0–91 years of age. From each geographic location, 2 independent sets of samples collected before and after the 2013 Australia PeV-A3 outbreak were used (Table 1). No ethics approval is required for anonymous use of biobank specimens in the Netherlands. Serum samples from the Netherlands in 2006–2007 came from a serum bank approved by the Medical Ethics Testing Committee of the Foundation of Therapeutic Evaluation of Medicines (ISRCTN 20164309). The institutional review board at the Children’s Mercy Hospital (Kansas City, Missouri, USA) determined that anonymous use of the Missouri samples was exempt from ethics approval. The human research ethics committee at Melbourne Health approved the use of Victoria serum samples and the human research ethics committee at Western Sydney Local Health District approved the use of New South Wales serum samples (LNR/17/WMEAD/279).

**Table 1:** Demographic information for study of parechovirus A3 neutralizing antibodies, Australia, the Netherlands, and United States*

Sample group	Institute	Sample type	No. (%) patients	Patient age, y	
				Mean	SD
Country (state) and years					
NL 2006–2007	RIVM	P	140 (11)	27.8	21.9
NL 2015–2016	AMC	R, S	140 (11)	27.8	21.5
USA (MO) 2012–2013	CMH	R	120 (9)	30.8	18.3
USA (MO) 2017	CMH, TMC	R	171 (13)	25.5	18.8
AUS (VIC) 2011–2012	VIDRL	R	138 (11)	26.5	19.9
AUS (VIC) 2015–2016	VIDRL	R	138 (11)	26.4	19.6
AUS (NSW) 2011–2012	WH, POW	R	185 (14)	26.1	23.2
AUS (NSW) 2015–2016	WH, POW	R	257 (20)	23.9	20.6
Sex†					
M			598 (46)	25.7	21.3
F			580 (45)	25.5	20.5
Age, y					
<1			148 (11)	0.4	0.3
1–2			52 (4)	1.8	0.6
3–4			41 (3)	3.8	0.6
5–9			120 (9)	7.2	1.5
10–19			220 (17)	15.8	2.7
20–29			184 (14)	24.8	2.9
30–39			172 (13)	34.2	2.8
40–49			162 (13)	44.6	2.8
50–59			89 (7)	54.8	3.2
60–69			62 (5)	64.3	2.8
>69			38 (3)	76.4	5.3
Total			1,288		

*AMC, Academic Medical Center; AUS, Australia; CMH, Children’s Mercy Hospital; NL, the Netherlands; P, population-based sampling; POW, Prince of Wales Hospital; R, residual serum from hospitalized patients and community; RIVM, National Institute for Public Health and the Environment; S, AMC staff; SD, standard deviation; TMC, Truman Medical Center; VIC, Victoria; NSW, New South Wales; VIDRL, Victorian Infectious Diseases Reference Laboratory; WH, Westmead Hospital. †Information on sex not available for US 2017 adult samples.

We tested the serum samples with a previously described neutralization assay (*8*). We serially diluted heat-inactivated serum samples and incubated them with chloroform-treated PeV-A3 strain isolated during the 2013 outbreak in Australia (GenBank accession no. KY930881) (*4*). We subsequently added LLCMK2 cells and incubated them for 7 days. We calculated neutralizing titers based on cytopathogenic effect using the Reed and Muench method and reported them as the reciprocal titers of serum dilutions exhibiting 50% neutralization (*9*). We considered an nAb titer of >1:8 to be positive; we used >1:32 as a secondary cutoff (*5*). We compared PeV-A3 nAb seroprevalence between the timepoints within each location using χ^2^ tests. We performed logistic regression to examine the association between seropositivity and location–timepoint (8 categories), gender (2 categories), and age (3 categories). We present 3 univariable models and 1 multivariable model including all 3 variables. We used the Kruskal-Wallis test with post hoc analysis and Bonferroni correction to compare the median nAb titers. In the statistical analyses, we excluded children <1 year of age because of the presence of maternal antibodies; we merged the remaining age categories into 3 groups.

**Figure 1:**
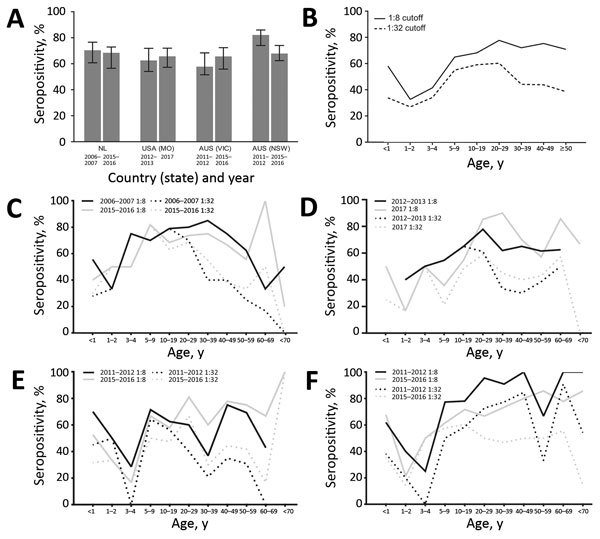
Parechovirus A3 (PeV-A3 neutralizing antibody (nAb) seropositivity, Australia, the Netherlands, and United States. A) Overall nAb seropositivity with associated 95% CIs. Infants <1 year of age were excluded from the analysis. Seropositivity rates between the timepoints within each location were compared by using χ^2^ tests. B) Overall age-stratified PeV-A3 nAb seropositivity, including infants <1 year of age. Seropositivity was determined as a nAb titer of ≥1:8 or ≥1:32. C–F) Age-stratified PeV-A3 nAb seropositivity in C) the Netherlands; D) Missouri, USA; E) Victoria, Australia; and F) New South Wales, Australia. Complete data used in this figure can be found in the Appendix (http://wwwnc.cdc.gov/EID/article/25/1/18-0352-App1.pdf). AUS, Australia; NL, the Netherlands; NSW, New South Wales; VIC, Victoria.

Overall PeV-A3 nAb seropositivity was similar across 3 locations: 71.1% (2006–2007) and 69.2% (2015–2016) in the Netherlands, 63.3% (2012–2013) and 66.5% (2017) in Missouri, and 58.5% (2011–2012) and 66.4% (2015–2016) in Victoria (Figure 1, panel A). In New South Wales, nAb seroprevalence was 82.9% in 2011–2012, whereas it was significantly less (68.6%) in 2015–2016 (p = 0.005; Figure 1, panel A). 

Age was a significant determinant of PeV-A3 nAb seropositivity, which increased from 32.7% in children 1–2 years of age to 65.0% in those 5–9 years of age and peaked at 77.7% in adults 20–30 years of age (Figure 1, panel B). nAb seropositivity decreased to 42.1% in persons >30 years of age when a titer cutoff >1:32, the level necessary for protection against disease (*5*), was used. Furthermore, we observed that only 33.8% of infants <1 year of age had an nAb titer >1:32 and were thus sufficiently protected by maternal antibodies (Figure 1, panel B). 

**Figure 2:**
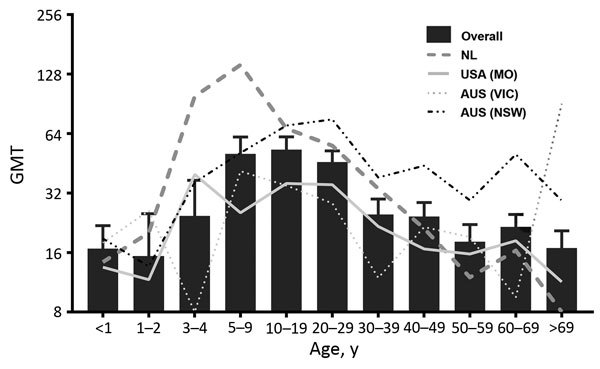
Age-associated GMTs of parechovirus A3 neutralizing antibodies, Australia, the Netherlands, and United States. Bars indicate overall GMTs (timepoints and locations merged); error bars indicate SDs. Lines represent GMTs in each location (timepoints merged). AUS, Australia; GMT, geometric mean titer; NL, Netherlands; VIC, Victoria; NSW, New South Wales.

We compiled age-stratified seroprevalences for each location and timepoint (Figure 1, panels C–F). The variables location–timepoint and age were significantly associated with seroprevalence in both univariable and multivariable regression models (p<0.002; Table 2). We did not detect sex-dependent differences (p = 0.309; Table 2). In line with the age-stratified seropositivity, the geometric mean titers (GMTs) declined steadily with age (Figure 2). Overall GMT peaked at 1:53 (SD 8.5) in the 10–19-year age group and decreased thereafter. Both children 1–5 years of age (p = 0.001) and adults >30 years of age (p<0.001) had significantly lower median titers than persons 6–29 years of age.

**Table 2:** Association between seropositivity and location–timepoint, gender, and age by univariate and multivariate logistic regression models in study of parechovirus A3 neutralizing antibodies, Australia, the Netherlands, and United States*

Parameter	Univariate model			Multivariate model	
	Odds ratio (95% CI)	p value		Odds ratio (95% CI)	p value
Location, years		**0.002**			**<0.001**
Netherlands, 2006–2007	1.124 (0.693–1.824)			1.078 (0.658–1.764)	
Netherlands, 2015–2016	1.026 (0.635–1.658)			0.963 (0.591–1.568)	
Missouri, USA, 2011–2012	0.790 (0.496–1.260)			0.725 (0.451–1.166)	
Missouri, USA, 2017	0.906 (0.585–1.403)			0.376 (0.189–0.748)	
Victoria, Australia, 2011–2012	0.644 (0.406–1.023)			0.642 (0.400–1.030)	
Victoria, Australia, 2015–2016	0.904 (0.563–1.451)			0.868 (0.536–1.406)	
New South Wales, Australia, 2011–2012	2.222 (1.354–3.647)			2.247 (1.358–3.718)	
New South Wales, Australia, 2015–2016†	1			1	
Sex‡		0.193			0.309
F	0.840 (0.646–1.092)			0.868 (0.661–1.140)	
M†	1			1	
Age, y		**<0.001**			**<0.001**
1–5†	1			1	
6–29	2.938 (1.954–4.419)			2.782 (1.821–4.251)	
≥30	3.248 (2.160–4.883)			3.081 (2.004–4.738)	

*Boldface indicates a statistically significant result. Seropositivity was determined as a neutralizing antibody titer ≥1:8. Children <1 years of age were excluded. †Reference category. ‡Information on sex not available for US 2017 adult samples.

## Conclusions

In this large seroepidemiological PeV-A3 study, we compared the nAb prevalence in populations from 4 distinct geographic regions. We report high and comparable PeV-A3 nAb seropositivity across all these regions. In agreement with the reports from Japan, the overall seroprevalence was 68.9%, suggesting widespread global circulation of PeV-A3 (*10**,**11*). Unexpectedly, the level of PeV-A3 humoral immunity in NSW was higher before the 2013 outbreak compared with 2–3 years after the outbreak. This suggests that PeV-A3 was already endemic in Australia before or during 2011–2012. Localized smaller PeV-A3 upsurges or variations in the proportion of samples originating from hospitalized patients versus the community may explain the observed difference between the earlier and later time periods.

Age-stratified PeV-A3 nAb seropositivity and GMTs suggest that the infection generally occurs in children <10 years of age, although nAb titers continued to increase in adolescent children. nAb titers decreased below the proposed level of protection in adults >30 years of age. Similar observations have been reported previously (*5**,**10**,**12*). This result is in contrast to results for PeV-A1, against which high nAb seropositivity rates are maintained in adults (*11*). The large proportion of seronegative persons and gradually declining GMTs in older age categories may indicate that widespread circulation of PeV-A3 has emerged fairly recently, as previously proposed (*13*), or that the immunity elicited in childhood is waning. Because the mean age of women at first birth in developed countries is high, we hypothesize that low nAb titers in women of childbearing age, and therefore the lack of adequate maternal antibody protection, contribute to the occurrence of PeV-A3 outbreaks in infants. Moreover, the 2013 Australia outbreak strain was recently described as a novel recombinant with the capsid-encoding region of the genome originating from a PeV-A3 strain collected in Japan in 2011 and the nonstructural region from an unknown origin (*4*). Preexisting serum antibodies recognizing epitopes in the PeV-A3 capsid maintain their ability to neutralize this strain, but this factor may represent a more virulent variant of PeV-A3.

This study has limitations. Cross-neutralizing antibodies resulting from exposure to other PeV genotypes may confound our findings. However, we have previously observed no evidence of PeV-A3 cross-neutralization by polyclonal and monoclonal antibodies elicited against PeV-A1 to 5 (*14**,**15*). Because we used anonymous serum samples from population-based sampling and residual serum collections, we could not relate the seroprevalence to cohort exposure history or etiologic information, and the varying sampling time periods prohibit us from making direct temporal comparisons between the locations.

Taken together, our results suggest that PeV-A3 circulation is widespread and that infection takes place in early childhood and adolescence. Nonetheless, PeV-A3 outbreaks occur regularly in young infants, and case numbers remain elevated in Australia (L. Caly, Doherty Institute, Melbourne, VIC, Australia, pers. comm. 2017 Oct 15). Why humoral immunity against PeV-A3 declines with age and what factors predispose neonates to severe PeV-A3 illness remain to be elucidated. Implementation of molecular PeV detection in routine diagnostics and continuous surveillance are warranted.

Appendix Additional information for study of parechovirus A3 neutralizing antibody seropositivity, Australia, the Netherlands, and United States.
