# Supplementary material for: Seroepidemiology of Parechovirus A3 Neutralizing Antibodies, Australia, the Netherlands, and United States
Source: Emerg Infect Dis. 2019 Jan;25(1):148–52. doi: 10.3201/eid2501.180352 (PMC6302606; doi:10.3201/eid2501.180352)
Supplement: Appendix — Additional information for study of parechovirus A3 neutralizing antibody seropositivity, Australia, the Netherlands, and United States. [file 18-0352-Techapp-s1.pdf]

Article DOI: https://doi.org/10.3201/eid2501.180352

Seroepidemiology of Parechovirus A3 Neutralizing Antibodies, Australia, the Netherlands, and United States

Appendix

Appendix Table. Total number of positive samples (for 1:8 and 1:32) by age category (in years), and numbers of samples for each location, timepoint, and age category.

Age, y	<1	1–2	3–4	5–9	10–19	20–29	30–39	40–49	50–59	60–69	≥70	Total
Overall												
Positive 1:8	86	17	17	78	150	143	124	122	60	46	28	871
Positive 1:32	50	14	14	66	130	111	76	71	34	29	10	605
Total	148	52	41	120	220	184	172	162	89	62	38	1288
Netherlands												
Total 2006–07	18	6	4	10	19	20	20	20	8	6	8	139
Total 2015–16	20	6	2	11	19	19	20	21	9	8	5	140
Missouri, USA												
Total 2012–13	0	5	4	11	20	18	21	20	13	8	0	120
Total 2017	16	6	4	14	33	34	20	20	14	7	3	171
Victoria, Australia												
Total 2011–12	20	2	7	14	16	20	19	20	13	7	0	138
Total 2015–16	19	3	6	12	19	21	20	18	12	6	2	138
New South Wales, Australia												
Total 2011–12	21	10	4	22	41	22	22	13	6	11	13	185
Total 2015–16	34	14	10	26	53	30	30	30	14	9	7	257
